# The Efficacy of Thai Herbal Prasaplai Formula for Treatment of Primary Dysmenorrhea: A Short-Term Randomized Controlled Trial

**DOI:** 10.1155/2016/2096797

**Published:** 2016-10-18

**Authors:** Manmas Vannabhum, Sirikan Poopong, Thanyarat Wongwananuruk, Akarin Nimmannit, Ueamphon Suwannatrai, Chongdee Dangrat, Angkana Apichartvorakit, Suksalin Booranasubkajorn, Tawee Laohapand, Pravit Akaraserenont

**Affiliations:** ^1^Center of Applied Thai Traditional Medicine, Faculty of Medicine Siriraj Hospital, Mahidol University, Bangkok, Thailand; ^2^Department of Obstetrics and Gynecology, Faculty of Medicine Siriraj Hospital, Mahidol University, Bangkok, Thailand; ^3^Office for Research and Development, Faculty of Medicine Siriraj Hospital, Mahidol University, Bangkok, Thailand; ^4^Department of Pharmacology, Faculty of Medicine Siriraj Hospital, Mahidol University, Bangkok, Thailand

## Abstract

This study aims to compare the efficacy of pain relief between a specific Thai herbal Prasaplai formula (PPF) and placebo in patients with primary dysmenorrhea. Forty women with primary dysmenorrhea symptoms were randomized into two groups. The experimental group received PPF capsules 1000 mg orally three times per day before meals for three days starting from the first day of menstruation. The placebo group received placebo as the same dose and time. Average pain intensity from the first day to the third day of cycle significantly decreased in both groups (*p* < 0.001), but with no statistically significant difference between groups. Using a pre- and posttreatment difference in NRS of at least 2, a greater proportion of patients in PPF group experienced pain relief compared to placebo during the first and second day of period. A greater proportion of PPF group also experienced no pain compared to the placebo group on day 1 and day 3 by using multidimensional scoring. The PPF demonstrates pain relief activity when used during menstruation in primary dysmenorrhea patients, with no adverse effects. However, further studies are needed in order to assess the value of PPF as a long-term prophylaxis for primary dysmenorrhea.

## 1. Introduction

Primary dysmenorrhea is the most common gynecological complaint in reproductive women. It is characterized by painful uterine contraction or menstrual cramps, occurring during menstruation without pelvic pathologies [1, 2]. Pain usually occurs intensely on the first or second day or the first 24–36 hours of menstruation [2]. In Thailand, the overall prevalence of dysmenorrhea in adolescents was 84.2%. Primary dysmenorrhea impacts daily activities, work, and academic performance [3].

Pathophysiology of primary dysmenorrhea is associated with the abnormal increase in prostaglandins secretion during menstruation. Prostaglandins are synthesized from arachidonic acid which is produced from phospholipids via the cyclooxygenase (COX) pathway [2]. The increase of prostaglandins during the menstrual phase has been shown to correlate with pain intensity and also increases uterine muscle contraction [2, 4, 5]. Pain relief is the main goal of treatment. Generally, primary dysmenorrhea is treated with nonsteroidal anti-inflammatory drugs (NSAIDs) such as mefenamic acid and ibuprofen [4, 6–9] despite the frequent adverse effect of gastrointestinal disturbance [7, 8, 10]. On the other hand, cyclooxygenase-2 (COX-2) inhibitors can also relieve pain without gastrointestinal side effects, but the price is relatively high [7, 11]. Traditional medicines present the alternative which have been used for many years globally [12]. Scientific studies support the use of herbal medicines as a viable option for the treatment of primary dysmenorrhea [13–17]. However, the ingredients and usage of these herbal recipes were different depending on traditional knowledge and beliefs.

Traditionally, Prasaplai in Thai traditional herbal medicine is used as prophylaxis for regulating menstrual flow. Thai traditional medicine theories believe that pain during menstruation occurs from imbalance of elements within the body. Plai is one of the Thai herbal formulas. It is used for pain relief and is able to restore body elemental balance. By clinical use in the Center of Applied Thai Traditional Medicine at Siriraj Hospital to control dysmenorrhea, the dosage of Prasaplai is reported to be 600 to 1000 mg three times a day before meals, 7 days before menstruation until the first day of menstruation. This regimen has been lacking in experimental trials to significantly prove its efficacy.

On the other hand, Plai in Prasaplai has been shown to also have an anti-inflammatory effect [18]. An in vitro study reported that Prasaplai recipe acts as COX inhibitors which can help relieve pain from the primary dysmenorrhea [18]. In human, a piloted study demonstrated that Prasaplai can reduce pain due to primary dysmenorrhea in patients [19]. Although Plai is always the key ingredient, differences in recipes exist due to local knowledge and practice in different regions of Thailand. For this research, we formulated PPF, a Thai herbal Prasaplai formula. So, the ingredients may be standardized. For the purpose of this paper, the Thai herbal Prasaplai formula will be called PPF, whereas other Prasaplai formulas will be simply referred to as Prasaplai. Siriraj Ayurvedic Clinic has been using PPF for the treatment of irregular menstrual cycle and relief of pain during menstruation for more than 30 years. Anecdotal claims of pain reduction have been reported, without scientific evidence.

When used for its anti-inflammatory and pain-relieving property, Prasaplai is taken at 1000 mg, three times a day before meals. This is the method proposed and approved by the National Drug List of Herbal Medicinal Products AD 2008 [20]. In a previous study by Sriyakul et al. [21], 400 mg Prasaplai capsule given three times a day for three days is compared with 500 mg mefenamic acid capsule. The Prasaplai group experienced similar pain relief to mefenamic acid group [21]. However, the pain is a condition which renders itself and may show significant result as placebo effect. Thus, in order to establish its true efficacy in relieving pain, a randomized, double-blind, placebo-controlled trial is needed. Also, Prasaplai is now used for its anti-inflammatory property. A higher dose should be tested and any possible adverse reaction should be ascertained. The primary objective of this study is to compare the efficacy of PPF versus placebo in primary dysmenorrhea patients. We used the dosage listed in the National Drug List of Herbal Medicinal Products AD 2008. The secondary objective is to determine side effects and user satisfaction with PPF.

## 2. Method

### 2.1. Study Participants

This was a randomized, double-blind, placebo-controlled trial, conducted at the Department of Obstetrics and Gynecology, Faculty of Medicine Siriraj Hospital, during February 2012 until March 2013. Inclusion criteria for participants were women aged 18 to 45 years, with regular menstrual cycles, who had been diagnosed with primary dysmenorrhea with menstrual pain scores higher than 5 out of 10 on Numeric Rating Scores (NRS) lasting for 3 months or experiencing moderate to severe pain intensity. The exclusion criteria were a history of allergy to ingredients contained in PPF and/or starch, placement of intrauterine devices, pregnancy, any gynecologic conditions, hormones used, breastfeeding period, and other medical history which can cause abdominal pain such as gall bladder, gallstone, dyspepsia, or irritable bowel syndrome. This study had been registered at clinicaltrials.gov, ID number NCT01598012. The registration was performed after the enrollment process but before randomization. The protocol was approved by the Siriraj Institutional Review Board (SIRB), Faculty of Medicine Siriraj Hospital, Mahidol University, Bangkok, Thailand (certificate of approval (COA) number Si 461/2011).

The participants were screened for symptoms of primary dysmenorrhea at the Gynecologic Endocrinology Unit, Department of Obstetrics and Gynecology, Siriraj Hospital. Primary dysmenorrhea was confirmed by exclusion of other pelvic pathologies through pelvic ultrasonography. All participants who met the inclusion criteria were enrolled and signed informed consent to participate in this study.

### 2.2. Sample Size

The estimate sample size was based on a previous study, studying the effect of an Iranian herbal drug on primary dysmenorrhea [14] where the difference in mean of pain reduction of herbal drug was 2.0 on the visual analogue scale (VAS), standard deviation was 2 with 5% type I error (2-sided test), and the power was 80%. The twenty participants by group were recruited with an additional 20% to account for loss of participants.

### 2.3. Intervention

Both PPF and placebo were produced at Herbal Medicines and Products Manufacturing Unit, manufactured under GMP by Ayurved Siriraj, Center of Applied Thai Traditional Medicine (CATTM), Faculty of Medicine Siriraj Hospital, Mahidol University, Thailand (GMP certified since 2009). PPF capsule contained 500 mg of PPF. Placebo capsule contained 500 mg of starch. Either PPF or placebo powder was filled in yellow opaque capsules. PPF has a distinct odor. In order to mimic smelling in the placebo, sponges containing PPF scent were packed together with both types of capsules. All bottles were labelled with the code which was known only by the manufacturers.

The randomization was done by software computer block randomization in which computer-generated codes were concealed in opaque envelopes. The experimental groups received twenty capsules of PPF 500 mg. The control group received twenty capsules of placebo 500 mg. All groups were instructed to take orally two capsules beginning on the first day of their menstruation and record pain score as NRS at this time, in this study defined as pretreatment pain score. Then, the dosage was changed to two capsules three times per day before meals for the first three days of each menstrual cycle. The participant had to record the average pain score as NRS on days 1, 2, and 3 during the experiment. Both groups were given ten tablets of mefenamic acid (500 mg) as rescue drug. After one hour of taking the experimental drug, if pain became unbearable, a tablet of mefenamic acid was allowed every 6 hours. Participants could stop after the pain was controlled. They were instructed not to take other medications during the study.

### 2.4. Outcome and Data Collection

The outcome measurements were recorded in self-diary record for one menstrual cycle. The pain intensity was measured using NRS and multidimensional scales, recorded after taking drugs on the first three days and before taking mefenamic acid. NRS is a 10-point scale, with 0 representing no pain and 10 representing maximum pain. The multidimensional scoring was used for the assessment of the severity of dysmenorrhea. Mild dysmenorrhea (pain score less than 4) could be defined as menstruation that is painful but seldom inhibits the women's normal activity and analgesics are seldom required. Moderate dysmenorrhea (pain score 4–6) is defined as pain affecting daily activity which required analgesics. Severe dysmenorrhea (pain score more than 6) is defined as pain which clearly inhibits activity and is poorly controlled by analgesics. The satisfaction (ranking score from 1 to 5, 1 = very dissatisfied and 5 = very satisfied) and opinion of participants were obtained on the follow-up day.

### 2.5. Statistical Analysis

All analyses were performed using PASW statistics 18.0 (SPSS Inc., Chicago, IL, USA). The descriptive statistic was used for demographic data, with satisfying results which were presented as means and standard deviations. The paired *t*-test and Mann–Whitney *U* test were used to compare the differences of mean reduction within group. The unpaired *t*-test was used to compare the differences of mean reductions between groups. Statistical significance was considered when *p* value was less than 0.05.

## 3. Results

**Figure 1:**
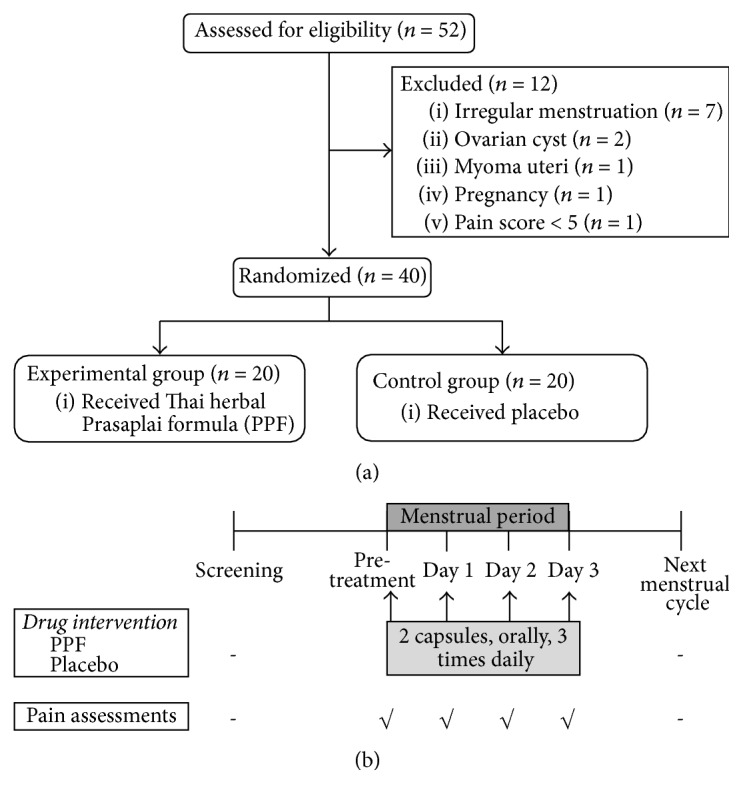
(a) Flow chart of the study. (b) Protocol of treatment and assessments. All interventions were started immediately after the first day of menstruation. Pretreatment is defined as the time of the start of menstruation before drugs are taken. Days 1, 2, and 3 are the days of menstruation after drugs are taken.

A total of 40 primary dysmenorrhea participants were allocated to this study as in Figure 1(a). The baseline characteristics and menstrual history of PPF and placebo group are shown in Table 1. Drug intervention was carried out 3 days continuously by starting from the first day of menstruation to day 3 (Figure 1(b)).

**Figure 2:**
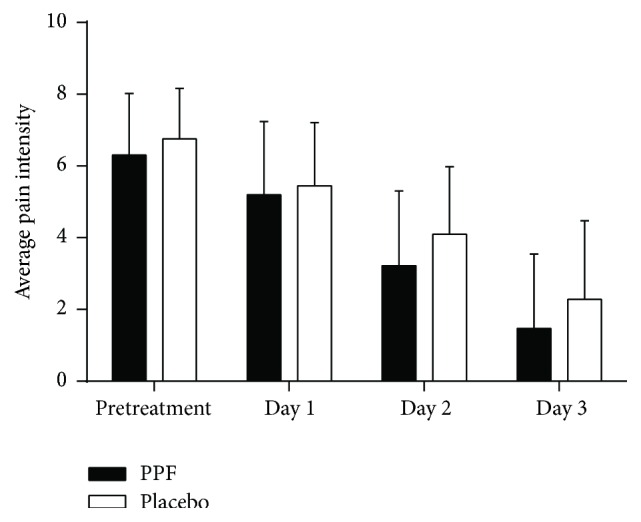
Pain scores before treatment on the last menstrual cycle and during pretreatment to day 3 between Thai herbal Prasaplai formula (PPF) and placebo groups. The average pain scores of the day are shown in mean + SD.

The results of pain scores between PPF and placebo groups during the last menstrual cycle and during pretreatment to day 3 are shown in Figure 2. The average pain intensity decreased significantly from day 1 to day 3 (*p* < 0.001) in both groups. The average pain scores between PPF and placebo showed no significant differences. Difference in pain scores before and after treatment in PPF group decreased more than in the placebo group, but with no statistical significance (Table 2).

**Figure 3:**
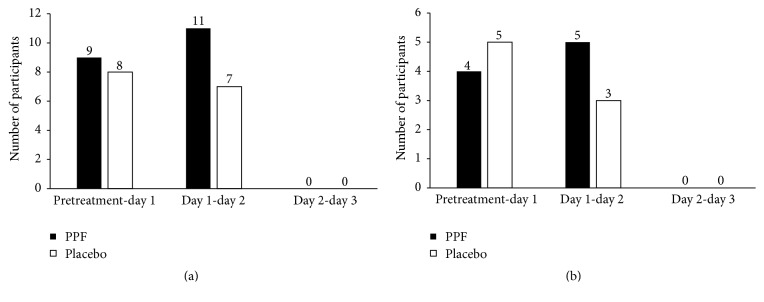
Number of participants with pain relieved where the criterion for pain relief was a pre- and postdifference in NRS of at least 2 between Thai herbal Prasaplai formula (PPF) and placebo groups. (a) All participants. (b) Participants who took mefenamic acid were excluded.

**Figure 4:**
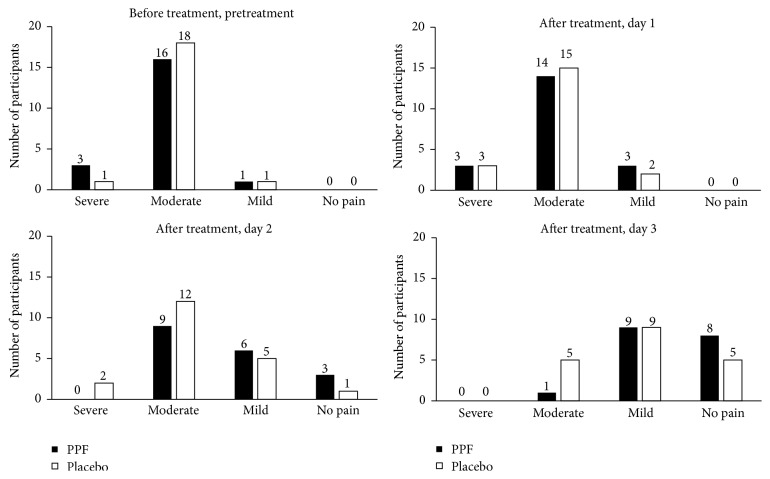
Severity of pain assessed with multidimensional scoring compared between Thai herbal Prasaplai formula (PPF) and placebo groups.

The criteria for pain relief included a pre- and posttreatment difference in NRS of at least 2 (Figure 3). PPF group had a greater proportion of participants with pain relief than the placebo group between pretreatment-day 1 and day 1-day 2. Overall, the number of participants who experienced pain relief in PPF group and placebo group was 20 and 15, respectively (Figure 3(a)). Among participants who did not need to take mefenamic acid as a rescue therapy, a greater portion of participants in PPF group reported pain relief compared with placebo group on day 1-day 2 (Figure 3(b)). In addition, the participants in PPF group used an overall average of 1.40 ± 1.96 tablets while the placebo group used 1.95 ± 2.40 tablets, though with no statistical significance (*p* value 0.407). Using the multidimensional scoring assessment, a greater proportion of the PPF group also showed no pain compared to placebo group on day 2 and day 3. There was no severe pain reported in the PPF group starting on day 2 (Figure 4). Overall, participants in both PPF and placebo groups reported equal satisfaction with the dosage form, scent, and method of administration.

## 4. Discussion

This is the first ever randomized control trial using PPF as a relief drug for menstrual pain as indicated by the National Drug List of Herbal Medicinal Products AD 2008 [20]. Although there is a significant placebo effect, the PPF group still demonstrated faster pain relief than the placebo group, as well as showing a tendency for reduced use of mefenamic acid.

Despite using a different drug formulation, dosage, and starting time for administration of Prasaplai, a pilot study of Prasaplai capsule compared to NSAIDs in treating primary dysmenorrhea found that Prasaplai taken 2-3 days before or during the menstrual period shows pain reduction similar to NSAIDs. This study also claims that Prasaplai can help regulate menstruation and achieve normal menstruum characteristics [19, 21]. In addition, a study in a rat model established the notion that Prasaplai has an antispasmodic effect which counteracts uterine contraction [22] as well as an in vitro study which reports the presence of anti-inflammatory prodrugs from Prasaplai extract, with no demonstrable cytotoxicity [23–26]. Therefore, this Thai traditional medicine has the potential to safely augment and expedite pain relief from dysmenorrhea in clinical practice. The previous studies about hormonal treatment for dysmenorrhea showed effective improvement of pain three to six months after use [27, 28]. In this study trial, results from only one cycle therapy are presented and the trend of pain relief score is revealed. Therefore, if the study protocol extends to more than one cycle, it might show a more significant pain score improvement.

In a previous clinical study by Sriyakul et al., 400 mg of Prasaplai capsule was administered three times a day for three days of menstruation for 6 cycles, which also showed no adverse effects both symptomatically and in laboratory studies including hematology, blood chemistry, liver function, and renal function [21]. Our study reports no serious adverse events. Only one case of aphthous ulcer presented in the PPF group which was not related to treatment. One case of abdominal flatulence is reported in the placebo group. Besides, the NSAIDs cost during primary dysmenorrhea treatment was lower in PPF group compared to placebo group. The cost of this traditional medicine is not high like the new generation of NSAIDs and there is no side effect on the gastrointestinal tract like in the past generation of NSAIDs.

According to Thai traditional medicine beliefs, Prasaplai is usually given as a prophylactic drug seven days before the start of menstrual period and over an extended period of time. On the other hand, our study uses PPF on a short-term basis for the immediate relief of menstrual pain. Consequently, the time interval may not be adequate for the drug to reach its potential. This difference in both the indication for use and the duration of use can account for the seeming ineffectiveness of PPF at relieving menstrual pain. Further studies designed to assess the efficacy of PPF when used as indicated by Thai traditional medicine recommendation should be carried out. Moreover, pain from dysmenorrhea naturally fluctuates from cycle to cycle; therefore, the pain assessment should take place over several cycles [29] to be accurate.

## 5. Conclusions

PPF demonstrates pain relief activity when used during menstruation in primary dysmenorrhea patients. It relieved pain on day 1 after it started. It has no side effect on the gastrointestinal tract like the past generation of NSAIDs and had no serious adverse effects. However, further studies are needed in order to assess the value of PPF as a long-term prophylaxis for primary dysmenorrhea as in the traditional recommendation.

## Figures and Tables

**Table 1:** Baseline characteristics and menstrual history of participants.

Characteristics	PPF (*N* = 20)	Placebo (*N* = 20)
Age (year)	22.5 ± 4.2 (min–max, 18–31)	26.6 ± 7.5 (min–max, 19–40)
BMI (kg/m^2^)	20.2 ± 2.1 (min–max, 18–26)	22.9 ± 4.6 (min–max, 18–32)
Marital status, *n* (%)		
Single	18 (90)	16 (80)
Married	2 (10)	4 (20)
Menarche (age)	12.7 ± 1.4	13.1 ± 1.2
Length of menstrual cycle (day)	27.8 ± 2.1	26.4 ± 6.0
Length of menstruation (day)	4.9 ± 1.1	5.3 ± 1.3
Previous treatments for pain		
Paracetamol	10 (50)	5 (25)
Mefenamic acid	10 (50)	10 (50)
Other drugs	0	2 (10)
No treatment	0	3 (15)
Pain severity before treatment		
Severe	3 (15)	1 (5)
Moderate	16 (80)	18 (90)
Mild	1 (5)	1 (5)

Data shown in mean ± SD or number (%).

**Table 2:** Mean difference in pain scores before and after treatment between Thai herbal Prasaplai formula (PPF) and placebo groups.

Day (before and after treatment)	Pain score by NRS Mean difference ± SD		*p* value
	PPF (*N* = 20)	Placebo (*N* = 20)	
Pretreatment	0.25 ± 1.11	0.20 ± 1.43	0.903
Day 1-day 2	2.11 ± 3.08	1.35 ± 1.82	0.315
Day 2-day 3	1.75 ± 1.64	1.83 ± 2.07	0.903
Day 1–day 3	3.86 ± 3.07	3.18 ± 2.57	0.460

Data are expressed in mean ± SD.

NRS: Numeric Rating Scores.

## Abbreviations

PPF: Thai herbal Prasaplai formula
NRS: Numeric Rating Scores
COX: Cyclooxygenase
NSAIDs: Nonsteroidal anti-inflammatory drugs
VAS: Visual analogue scale
CTTM: Center of Applied Thai Traditional Medicine.
